# Location, location: understanding the metastatic microenvironment

**DOI:** 10.1002/1878-0261.13492

**Published:** 2023-07-29

**Authors:** Neta Erez

**Affiliations:** ^1^ Department of Pathology, Faculty of Medicine Tel Aviv University Israel

**Keywords:** metastasis, metastatic niche, microenvironment, organ‐specific

## Abstract

Mortality from cancer is almost exclusively a result of tumor metastasis. Since advanced metastatic cancers are incurable, understanding the biology of tumor metastasis is one of the most significant challenges in cancer research today. A large body of research had established the central role of the microenvironment in facilitating tumor growth. However, the role of the metastatic microenvironment in supporting the multistage process of metastasis is still largely unresolved. To thrive at the metastatic site, disseminated cancer cells must adapt to distinct organ‐specific microenvironments that exert unique cellular and molecular interactions to oppose or support the growth of metastatic cancer cells. Understanding these intricate interactions is key to the development of effective therapeutic strategies that may prevent metastatic relapse.

## Graphical abstract

Understanding the biology of tumor metastasis is crucial to combat cancer mortality. The tumor microenvironment at metastatic organs plays a central role in enabling or opposing metastatic relapse. The interactions of the metastatic TME with disseminated cancer cells are organ‐specific, sex, and age‐dependent. Understanding these organ‐specific interactions is key to the development of effective therapeutic strategies.

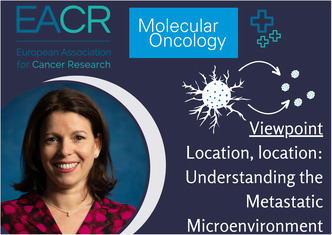

**Abbreviations**
ECM: extracellular matrix
TME: tumor microenvironment

Tumors are more than a collection of cancerous cells. Extensive research has led to the understanding that tumors are better described as multicellular organs or evolving ecosystems, composed of multiple types of stromal and immune cells, as well as extracellular matrix (ECM) components, collectively referred to as the tumor microenvironment (TME). The TME and its intricate interactions with cancer cells are a crucial player in all stages of tumorigenesis, progression, and response to therapy [1].

Most of our knowledge of the interactions between cancer cells and the surrounding immune and stromal cells stems from studies of primary tumors in mouse models and in patient samples [2]. However, the main cause of cancer mortality in most cancer types is tumor metastasis to distant organs.

Metastasis is a multistage process. In many tumor types, there is a long temporal lag (months to decades) between when malignant cells arrive in ectopic locations and their acquisition of capabilities that allow organ colonization [3]. Since advanced metastatic disease is still incurable, reaching a comprehensive understanding of the mechanisms underlying the early stages of the metastatic process is one of the most significant and urgent quests in cancer research today. Moreover, it is also an essential prerequisite for the discovery of novel therapeutic targets.

In patients, the early stages of metastasis, between resection of the primary tumor and diagnosis of clinically evident metastasis, are currently a ‘black box’ since micro‐metastatic lesions are undetectable, limiting our ability to curb metastatic relapse. Importantly, while the initial stages of carcinogenesis require multistage genetic changes to render normal cells cancerous, the disseminated cells that reach distant organs are already fully transformed and have also acquired invasive capacities. Nevertheless, metastatic relapse is often considerably delayed after the resection of primary tumors. One of the reasons for this interval is that in order to thrive at the metastatic site, disseminated cancer cells must adapt to a new, and sometimes hostile, organ‐specific microenvironments and acquire the capability to reprogram stromal cells in their new microenvironment to support their growth. However, our understanding of metastatic microenvironments is still limited.

Similar to the primary tumor microenvironment, the metastatic tumor niche is composed of various stromal and immune cell types that nurture and support the tumor cells. Moreover, the interactions of cancer cells with the TME at different metastatic sites have shown to precede metastases formation. Systemic signaling from the primary tumor instigates changes in the cell milieu, activation status, and ECM composition of cells at the future metastatic site, known as the formation of a hospitable premetastatic niche [4]. Once cancer cells have disseminated and reached metastatic sites, their interactions with the local TME may instruct their dormancy, rather than growth, thus determining the lag prior to clinically evident metastatic relapse [5]. Changes in this delicate balance of signaling with the local TME may lead to activation of tissue repair and inflammatory pathways that stimulate cells in the TME to enable the awakening of dormant cells and to support their growth [6, 7].

Importantly, each metastatic microenvironment exerts specific functions that support or oppose colonization by disseminated tumor cells [3, 8]. Indeed, the molecular interactions required to enable growth in, for example, bone, liver, brain, or lungs are distinct and require activation of organ‐specific adaptation mechanisms in cancer cells. Moreover, organ‐specific microenvironments may instruct common adaptation mechanisms in different cancer types [9]. Therefore, understanding the distinct organ‐specific mechanisms that enable metastatic growth is of crucial importance.

Interestingly, while the hallmark principles that govern the interactions of cancer cells with the TME are similar between cancer types, locations, and stages [2], the specific pathways that play a role in each metastatic organ are location‐dependent and often represent hijacking of pathological features characteristic of specific organs. For example, IL‐33, known to play a role in inflammatory lung diseases related to type‐2 immunity, is also important in facilitating lung metastasis [10]. In bone metastasis, NG2^+^ bone mesenchymal cells that participate in bone repair following fractures are hijacked to facilitate bone colonization by disseminated cancer cells [11]. Another demonstration of the importance of adapting to each metastatic TME is evident from observations that metastatic cancer cells express genes that mimic functional cells in the metastatic organ. For instance, both breast and prostate cancer cells (that often metastasize to bones) express osteoblast markers, a phenomenon known as osteomimicry. Brain mimicry was also observed in brain metastasizing cancer cells [12]. These examples (and many others) further emphasize the importance of studying the unique characteristics of each metastatic TME.

**Fig. 1:**
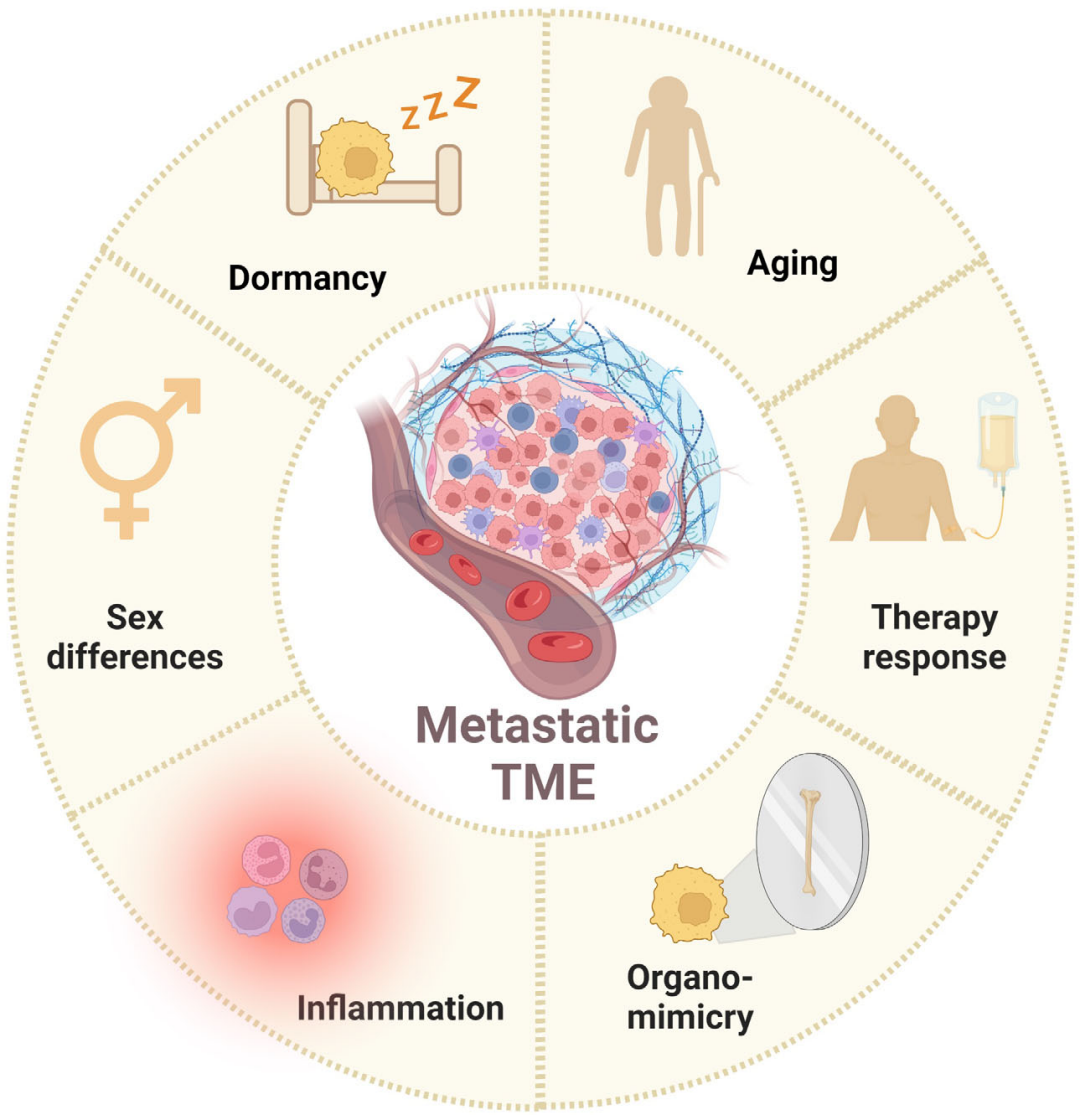
Metastatic TME plays a central role in facilitating or restraining metastatic relapse. The interactions of the TME with disseminated cancer cells are organ‐specific, sex and age‐dependent, and can modulate tumor dormancy, organo‐mimicry, and therapy response. Created using Biorender.com.

## So how can we approach this important task in a better and more relevant way?

While our ability to scrutinize the early stages of metastasis in patients is limited, well‐designed mouse models can provide us with valuable knowledge. Since metastasis is a multistage process involving the immune system, blood vessels, stromal cells, and ECM, it is crucial to study these processes in immune‐competent mice and, whenever possible, using models of spontaneous metastasis [13]. Moreover, studying the metastatic TME in comparison with organ‐specific pathologies may provide us with useful insights as we could repurpose existing treatments targeting organ‐specific pathways that may be hijacked in metastasis. Another emerging crucial issue to address is how sex differences in the TME affect metastasis. Although epidemiological studies indicate that cancer metastasis, outcome, and response to therapy are significantly influenced by sex [14], this important parameter has been largely ignored. In the coming years, we should increase our efforts in investigating the differences between TMEs in males and females to understand how sex affects tumor metastasis, as well as therapy response and resistance. In addition, when studying the metastatic microenvironment, we should consider the effect of aging: it is now clear that the aging TME is profoundly different in the constraints and interactions with disseminated cancer cells [15], which should be taken into consideration when designing experiments and interpreting data (Fig. 1).

The combined information from suitable mouse models, together with validation of findings in human metastasis specimens when available, will hopefully lead to discovery of pathways and interactions of disseminated cancer cells with organ‐specific metastatic TMEs. This knowledge should be harnessed for the stratification of cancer patients according to their metastatic risks, and the design of strategies aimed at impeding metastasis in specific organs. The development of such novel therapeutics, or repurposing of existing drugs, will hopefully enable chronic treatments administered following resection of the primary tumor that will prevent, rather than try to cure metastatic disease.

## Conflict of interest

The author declares no conflict of interest.
